# VSD应用于胸部手术后感染防治体会

**DOI:** 10.3779/j.issn.1009-3419.2018.04.25

**Published:** 2018-04-20

**Authors:** 继龙 马, 静 赵, 启州 柏, 生亮 贺, 珺 于, 云久 苟

**Affiliations:** 1 730000 兰州，甘肃中医药大学 Gansu University of Traditional Chinese Medicine, Lanzhou 730000, China; 2 730000 兰州，甘肃省人民医院 Gansu Provincial Hospital, Lanzhou 730000, China; 3 730000 兰州，兰州市第一人民医院 The first people's Hospital of Lanzhou, Lanzhou 730000, China

**Keywords:** VSD, 引流, 术后, 伤口感染, 胸外科, VSD, Drainage, Postoperative, Wound infection, Thoracic surgery

## Abstract

**背景与目的:**

手术切口感染是胸外科术后常见并发症之一，其危害与感染程度、部位等相关，轻者致局部疼痛、住院时间延长、费用增加，重者可导致严重感染，甚至感染性休克、危及生命。因此，妥善处理切口感染，有利于促进恢复、降低疾病负担、奠定进一步治疗良好基础。切口感染传统外科处理措施包括彻底引流、加强换药、使用抗生素等，存在治疗过程长、治疗效果不确切等不足。本研究对我科6例胸部手术术后发生感染患者尝试性使用负压封闭引流装置（vacuum sealing drainage, VSD）的经验进行总结，以期改进传统应对患者胸部手术术后感染的处理模式。

**方法:**

对我院近一年来出现胸部手术术后切口感染或手术切口瘘的患者相关临床数据进行回顾和总结，选择了其中6例使用VSD材料治疗术后感染的患者，对其使用VSD处理的过程和最终临床结果进行总结讨论。

**结果:**

本研究中所有患者在使用VSD后6 h-10 h内发热、伤口渗出症状消失。7天-10天后拔除引流装置，5例患者创面感染情况明显改善，伤口分泌物消失，手术切缘肉芽组织生长良好，二期手术关闭胸腔和皮肤。1例患者感染严重，去除VSD后分泌物仍较多，效果不明显，再次放置VSD装置，7天后去除VSD装置，患者手术切口无渗出，肉芽组织生长良好，二期手术关闭胸腔和皮肤。所有6例患者最终感染症状缓解，症状改善，手术切口愈合良好出院。2例食管癌患者中，平均手术时间427.5 min，术后平均住院天数40天，术后平均换药次数8.5次，住院期间平均总花费111, 893.47元；4例慢性脓胸患者中，平均手术时间192.5 min，术后平均住院天数27.75天，术后平均换药次数5.5次，住院期间平均总花费48, 237.71元。

**结论:**

VSD在处理胸外科手术术后切口感染患者中效果良好，减少了患者的痛苦和负担，保证了发生术后感染患者的生活质量。

## Abstract (en)

**Background and objective:**

Surgical site infection is one of the common postoperative complications of thoracic surgery, and its harm is related to infection degree and location. Light causes local pain, prolonged hospitalization and increased cost. Severe infection can lead to severe infection, even septic shock and life-threatening. Therefore, proper treatment of incision infection can help to promote recovery, reduce the burden of disease and lay a good foundation for further treatment. The traditional surgical treatment of wound infection includes thorough drainage, intensive dressing change and antibiotic use. There are many shortcomings such as long treatment process, ineffective treatment effect and so on. The experience of using vacuum sealing drainage (VSD) in 6 cases of postoperative infection patients in our department is summarized in order to improve the traditional treatment of postoperative infection in patients after thoracic surgery.

**Methods:**

The clinical data of patients with postoperative incision infection or fistula after thoracic surgery in our department were reviewed and summarized. 6 patients treated with VSD material for postoperative infection. The process and final clinical results of them were summarized and discussed.

**Results:**

In this study, fever and wound exudation disappeared within 6 h-10 h after VSD use, 5 cases of wound infection were obviously improved, the secretion disappeared and the granulation tissue grew well at the cutting edge of the operation, the second stage operation was performed to close the chest and skin. One patient was seriously infected, and the secretion was still more after VSD removal, reposition VSD device next time, the VSD device was removed 7 d later. The wound infection were obviously improved, the secretion disappeared and the granulation tissue grew well, close the chest and skin second stage. In all 6 patients, the symptoms were relieved, the symptoms improved and the surgical incision healed well. In 2 patients with esophageal cancer, the average operation time was 427.5 min, the average hospitalization time was 40 d, the average number of times of dressing change was 8.5, the average total cost during hospitalization was 111, 893.47 yuan patients with chronic empyema, the average operation time was 192.5 min. The average hospital stay was 27.75 days, the average number of times of dressing change was 5.5, and the average total expenditure during hospitalization was 48, 237.71 yuan.

**Conclusion:**

VSD has a good effect on the treatment of postoperative incision infection patients in thoracic surgery. It can reduce the pain and burden of patients and ensure the quality of life of postoperative infected patients.

手术切口感染（surgery site infection, SSI）是外科术后最常见的术后并发症之一，广泛发生于多种外科手术术后，包括普外科、烧伤科、胸外科及泌尿外科手术^[1]^。SSI的发生可能与手术时间长、手术切口大、手术切口吻合瘘、术后缝合过程中遗留的残腔以及手术部位本身存在的感染等诸多因素相关^[1, 2]^。研究^[3]^表明我国SSI发生率排名前3位的科室分别是骨科（5.48%）、胸外科（4.19%）以及泌尿外科（2.93%）。骨折患者在术前多已存在一定程度的感染，而胸部手术因为其手术部位特殊，手术操作时间长，发生感染的几率仅次于骨科^[4]^，而胸部手术涉及到心脏、肺以及大血管等在内的诸多重要脏器和组织，一旦发生SSI，后果严重，如果感染扩散至全身甚至还会引起患者严重的肝肾功能严重损害而最终导致死亡。

传统胸外科手术术后发生SSI后的治疗主要包括充分引流、彻底清除坏死组织和感染物、加强抗生素的使用以及多次换药等^[5]^。虽然传统多次换药的方法对患者手术切口感染的治疗有一定效果，但其起效慢，所需时间长，安全性较差且效果不确定，同时对患者造成了较大的痛苦和经济负担，而且长期大量的抗生素使用易造成抗生素滥用，最终处理效果并不理想^[6]^。

随着近年来医疗技术和新材料的不断进步与发展，负压封闭引流装置（vacuum sealing drainage, VSD）逐渐应用于SSI。其目前主要广泛应用于烧伤科和骨科，效果显著^[7, 8]^。本文作者通过文献检索后未发现关于VSD在胸外科SSI的处理的相关报道。在胸外科日常手术中，最常发生术后切口感染的是慢性脓胸纤维板剥脱术，两切口和三切口食管癌根治术。本研究报道了2016年6月-2017年6月我院胸部手术发生SSI后使用VSD的6例患者术前术后的相关情况，以期对胸部手术SSI中使用VSD的经验进行分享。

## 1 资料和方法

### 1.1 一般资料

本研究纳入2016年6月-2017年6月我院6例术后感染患者，其中食管癌2例，均为男性，平均年龄59岁，行颈胸腹联合三切口食管癌根治术。脓胸患者共4例（右侧慢性脓胸3例，左侧慢性脓胸1例），其中3例女性1例男性，平均年龄44.25岁；行开胸直视下纤维板剥脱术。6例手术均由同一外科小组完成（患者具体一般资料见表 1）。

**1:** 患者一般资料 Patients characteristic

	Esophageal cancer (*n*=2)	Pyothorax (*n*=4)
Age	59(58-60)	44.25(34-49)
Gender		
Male	2	1
Female	0	3
Surgical method	Resection of esophageal carcinoma	Decortication
Operationtime(min)	427.50(425.00-430.00)	192.50(185.00-200.00)
Postoperative hospitalization days(d)	40(37-43)	27.75(25-33)
Postoperative times of dressing change	8.5(7-10)	5.5(4-8)
Cost(CNY)	111, 893.47(100, 587.45-123, 199.49)	48, 237.71(46, 282.53-50, 122.33)
a (b-c): mean (minimum-maximum).		

### 1.2 手术方式

食管癌：2例食管癌患者均采用颈、胸、腹联合治疗，术中清扫颈、胸、腹三野淋巴结，颈部吻合食管。右侧肋间、腹中央切口约15 cm，颈部切口约10 cm，平均手术时间约7 h。左/右慢性脓胸：左、右侧慢性脓胸患者在相应病变侧肋间切开暴露胸腔，切口长约15 cm，术中彻底剥离纤维板，平均手术时间约3 h。

### 1.3 感染及处理过程

所有6例患者在发现手术切口发生感染或瘘前均有发热症状出现，当患者出现体温升高并且物理降温无效时，给与患者手术切口换药并观察患者手术切口，其中1例患者切口出现切口渗液，其余5例患者手术切口干燥无渗液。6例患者均给予药物退热24 h后症状无改善，5例无渗液患者手术切口纱布全部浸湿，给予换药。所有患者的症状在重复换药一天后无明显好转。遂在我院烧伤科的协助下放置VSD。

### 1.4 VSD处理方式

对术后出现顽固性发热的患者，检查其血常规结果，给予切口换药、挤压、对渗出物进行细菌培养，根据患者白细胞计数、细菌培养结果和患者临床症状判断其是否发生术后切口感染。对发生术后切口感染的患者，通过对患者状态进行评估，对感染情况严重或可能出现吻合口瘘的患者一般选择放置VSD。由患者的主治医生与患者和其家属进行沟通，对其优劣及可能出现的后果进行详细说明，最终由患者及其家属沟通后自愿决定处理方式并签署相关知情同意书。

在使用VSD之前，首先完全打开感染的手术切口，彻底清除坏死组织，然后将大小可与切口匹配的聚乙烯泡沫完全填塞感染切口，然后用生物透性粘贴材料进行覆盖，薄膜覆盖范围应超过创缘5 cm以防漏气。两端分别与引流管相连，一端用生理盐水冲洗，另一端则用负压吸引装置吸引。负压吸力（-125 mmHg--250 mmHg）。装置连接完成，引力管固定妥当后，打开负压吸引阀门，聚乙烯泡沫材料和生物膜敷料明显塌陷则说明装置密封良好，不存在漏气，负压吸引效果满意；反之则需调整至满意为止。在负压吸引过程中给与抗生素抗感染治疗，严密观察患者生命体征和实验室检查结果。通常在7天-10天后去除VSD装置，如果患者手术切口肉芽组织生长良好、感染情况消失则对切口进行二期缝合。对去除VSD装置后手术切口组织不新鲜或者感染情况仍然存在的患者，需再次放置VSD，待感染控制后二期缝合切口。

## 2 结果

本研究中所有患者在使用VSD后6 h-10 h内发热、伤口渗出症状消失，7天-10天后去除VSD装置患者，5例创面感染情况明显改善，分泌物消失，手术切缘肉芽组织生长良好，二期手术关闭胸腔和皮肤。1例患者感染严重，去除VSD后分泌物仍较多，效果不明显，再次放置VSD装置，7天后去除VSD装置，患者手术切口无渗出，肉芽组织生长良好，二期手术关闭胸腔和皮肤。所有6例患者最终感染症状缓解，症状改善，手术切口愈合良好出院。

本研究中2例食管癌患者中，平均手术时间427.5 min，术后平均住院天数40天，术后平均换药次数8.5次，住院期间平均总花费11, 893.47元；4例慢性脓胸患者中，平均手术时间192.5 min，术后平均住院天数27.75天，术后平均换药次数5.5次，住院期间平均总花费111, 893.47元（见表 1）。

## 3 讨论

VSD作为一种新型的生物材料，广泛应用于烧伤科和骨科皮肤移植和皮瓣移植以及术后发生SSI患者的处理过程中^[9]^，但通过检索相关文献后发现目前并无VSD在胸部外科手术SSI处理中应用的报道。本研究对近一年来我科发生SSI后使用VSD装置处理的6例患者的发病过程和处理过程进行了总结描述，6例术后感染患者中，5例在首次使用VSD后感染症状得到控制，二期缝合后出院。1例在第一次使用VSD后，效果不明显，切口愈合不理想，仍有少量渗出，再次放置VSD后，感染症状得到控制。二期清创缝合切口后出院。

术后感染或瘘是所有外科手术术后最常见的并发症，胸外科手术中，胸膜纤维板剥脱术、两切口食管癌根治术和三切口食管癌根治术是最容易发生术后切口感染的三种手术。慢性脓胸患者纤维板形成过程中，感染因素和免疫机制互相对抗，从而产生胸腔的脓液，逐渐形成纤维组织板，因此发生脓胸形成纤维板的患者身体素质较好，胸壁较厚，皮下肌肉和脂肪层较厚，而加以患者本身患有感染、手术切口大、手术时间长，因此，患者术后极容易发生术后感染^[4]^。而食管癌两切口和食管癌三切口手术时间长，切口部位多，术中需要进行至少两次体位变换，术中食管吻合过程中又容易接触到消化道内的污染物以及术后存在发生吻合口瘘的机会，这都容易造成食管癌术后的感染^[10]^。

胸部手术较其他部位的手术有其特殊性，正常人体的呼吸功能主要由患者肋间肌肉和膈肌的运动调节胸腔负压完成。胸外科手术中经常需要对患者进行单腔气管插管和肺组织萎陷，因此术后我们需要鼓励患者咳嗽以促进胸腔积液积气随引流管排出，进行呼吸训练以促进患者肺复张。对于术后发生感染或吻合口瘘的患者，我们传统的处理方法是将患者手术切口完全打开，彻底清除坏死的组织，多次换药以及大剂量抗生素治疗。传统换药的方法破坏了患者胸腔的负压和密闭性，影响患者术后肺的复张和术后手术部位残液及坏死物质的排出。而至少每日一次的换药过程对患者手术切口不断刺激，使患者切口疼痛难忍，患者术后咳嗽困难，不利于患者术后肺组织的复张和手术切口的愈合。如果患者肺不能完全复张，容易引起术后肺炎甚至肺组织完全失去功能，后果严重^[4]^。

1977年Morykwas等^[11]^首先对真空负压吸引处理手术切口的方式进行了报道。此后，VSD装置逐渐被应用于普通外科和骨科等科室的术后感染切口^[9, 12, 13]^。普外科手术临近肠道，容易发生术中污染和术后吻合口瘘，导致感染；开放性骨折患者骨端外露，大部分患者骨折初期就已经发生污染，尽管术中对伤口进行消毒和处理，但术后发生感染的可能性仍然较大。VSD包括用于填塞伤口的聚乙烯泡沫材料和用以覆盖患者切口的粘性敷料，以及冲洗管和负压引流管。泡沫材料可以剪切成任意形状，可以充分填塞创口；粘性材料可以完全覆盖创口，使创口完全密闭；而独立的冲洗管和引流管可以无间断的对患者手术切口内的坏死物质和分泌物进行引流，完全控制患者手术切口的感染^[14, 15]^。

VSD装置通过适当的负压吸力，使作用局部与周围组织表面产生压力差促使创面血流灌注，增加毛细血管壁的开放，改善切口部位组织的氧供，有利于各种修复细胞增殖和发挥功能，促进肉芽组织的生长^[16]^；同时，吸引装置可以将手术切口附近甚至更大范围内的血液中的抗生素吸引到手术切口附近，使抗生素重点作用于感染部位，提高了抗生素的使用效率，避免了感染患者术后抗生素的滥用，也缩短了患者住院时间。Moues等^[17]^的研究对比局部负压治疗与传统方法对严重创伤的治疗效果，结果发现采负压吸引可促进伤口创面更快愈合，同时保证SSI患者术后的生活质量，并发症发生率更低。

对于胸外科手术中的SSI来说，VSD装置还有更加重要的作用。VSD装置除了通过聚乙烯材料对创口完全填塞，生物粘膜完全覆盖感染部位切口，将切口和外界完全隔离，避免切口再次被外界因素污染的同时VSD还可以维持胸腔的负压，使感染对肺呼吸功能的影响降到最低，避免术后肺炎等严重并发症的发生，同时吸引管可以无间断的排出胸腔内的坏死物质和残留液体，避免术后多次换药，减轻患者痛苦，加强患者术后咳嗽，有利于患者术后痰液的排出和肺复张。相关文献^[18]^报道，与传统换药相比，VSD可使创面愈合率提高61%，治疗成本（包括材料成本和护理费用）降低38%，减轻了患者家庭的经济负担。

尽管相比于传统多次换药，VSD的使用在处理SSI的过程中有着较大的优势，但在使用操作的过程中仍不可大意。要时刻注意观察引流装置的密闭性，如果漏气，会使负压下降，不能充分吸引渗出液及坏死组织，由于积液增多，可以堵塞导管，加速细菌繁殖，进而加重感染。同时，VSD使用过程中的压力选择也十分重要，压力过高会影响皮肤血供及供养，过低会影响引流效果，因此VSD负压封闭引流术后的观察及护理工作非常重要。

综上所述，对于胸部手术术后发生感染的患者，VSD负压引流技术不仅缩短了患者术后感染治疗的疗程，缩短治疗时间，减少治疗药物使用剂量，避免抗生素的滥用，也保证了手术切口部位皮肤的愈合的完整和美观。同时在胸外科SSI中，VSD还可以模拟皮肤的厚度和胸腔的负压，隔离感染切口，避免二次感染，维持胸腔负压，减轻患者痛苦，减轻患者术后并发症的发生率，相较于传统换药的处理方式，VSD在胸外科术后感染的处理上有着更明显的优势。
